# Epistemic solidarity in medicine and healthcare

**DOI:** 10.1007/s11019-022-10112-0

**Published:** 2022-08-31

**Authors:** Mirjam Pot

**Affiliations:** Department of Political Science, University of Vienna, Vienna, Austria

**Keywords:** Solidarity, Relationality, Justice, Injustice, Social epistemology, Doctor–patient encounters, Health data sharing

## Abstract

In this article, I apply the concept of solidarity to collective knowledge practices in healthcare. Generally, solidarity acknowledges that people are dependent on each other in many respects, and it captures those support practices that people engage in out of concern for others in whom they recognise a relevant similarity. Drawing on the rich literature on solidarity in bioethics and beyond, this article specifically discusses the role that *epistemic* solidarity can play in healthcare. It thus focuses, in particular, on solidarity’s relationship with justice and injustice. In this regard, it is argued (1) that justice and solidarity are two equally important and complementary values that should both be considered in healthcare practices and institutions and (2) that solidarity often arises in unjust situations and can be a means to bring about justice. I transfer these ‘general’ insights about solidarity to knowledge practices in healthcare and link them to the discussion about epistemic injustices in healthcare and how to overcome them. I argue that epistemic solidarity can play an important role in overcoming epistemic injustices as well as—and independently from its contribution to justice—in knowledge production in medicine more generally. To demonstrate how epistemic solidarity can add to our understanding of collective knowledge practices, I discuss two examples: patients sharing their medical data for research purposes and healthcare professionals’ engagement with patients to better understand their afflictions.

## Introduction: epistemic injustice in healthcare

In her seminal work on the topic, Miranda Fricker defines epistemic injustice as ‘a wrong done to someone specifically in their capacity as a knower’ (Fricker 2007, p. 1) and suggests differentiating between two forms of epistemic injustices: testimonial and hermeneutical injustice. Applying the concept to the context of healthcare, Havi Carel and Ian J. Kidd speak of ‘pathocentric epistemic injustice’^1^ to address the epistemic injustices that patients may experience (Carel and Kidd 2014) ; Carel and Kidd 2017; Kidd and Carel 2017; Kidd and Carel 2018). For healthcare, they specify that the first form of epistemic injustice—testimonial injustice—occurs when patients’ testimonies about their illness experiences are devalued because of particular stereotypes associated with patients. Examples of this type of injustice are patients not being granted enough time to speak during consultations, patients not being listened to, and what they say not being taken seriously or considered a relevant epistemic contribution. Instances such as these qualify as testimonial injustices if they are based on stereotypes about patients whereby healthcare providers consider their patients ‘cognitively unreliable, emotionally compromised, or existentially unstable in ways that render their testimonies and interpretations suspect’ (Carel and Kidd 2014, p. 530).

The second type of epistemic injustice, hermeneutical injustice, occurs when people face unfair disadvantages in making sense of and expressing their experiences because the ‘collective interpretive resources’ (Fricker 2007, p. 1) required to do so are unavailable. Hermeneutical injustice means that the necessary concepts to grasp and communicate particular social experiences are missing. Fricker (2007) gives the example of women who have experienced sexual harassment: Before the term ‘sexual harassment’ was part of the common lexicon, women who experienced harassment also tended to experience hermeneutical injustice because the lack of a term specifying the transgression made it difficult for them to make sense of their experiences and communicate them. Kidd and Carel (2017, p. 185) contend that patients often experience hermeneutical epistemic injustices because ‘ill persons typically have non-dominant hermeneutical resources that are not recognised or respected by the epistemically dominant healthcare professions, but which are essential to the understanding of at least certain aspects of the experience of illness.’ This means that when patients make sense of and describe their illness, they usually do this in lay terms. In the medical context, however, information conveyed in lay terms often ‘counts less’ than information communicated in the biomedical terminology that healthcare professionals use. The hermeneutical injustices patients may suffer are grounded in the fact that although their experiences are an critical component of fully grasping disease, they are often not recognised as epistemically valuable because they are difficult to reconcile with biomedicine, the dominant epistemic and conceptual framework in healthcare.

We can understand testimonial injustice as relating to *who* says something (for example, a patient) and hermeneutical injustice as relating to *how* something is said (for example, in lay terms). Medicine and healthcare are prone to both forms of pathocentric epistemic injustices—which are often intertwined—due to the status of biomedicine, which favours scientific knowledge over patients’ experiential knowledge and doctors’ detached third-person accounts over patients’ first-person testimonies. Epistemic injustices are closely connected to power dynamics, which are evident not only in the power asymmetry between doctors and patients but also in the legitimacy assigned to different forms of knowledge.

At the level of healthcare encounters, the concept of epistemic injustice has been discussed in connection with manifold issues such as chronic fatigue syndrome (Blease et al. 2017), chronic pain (Buchman et al. 2017), children’s testimony (Carel and Györffy 2014), mental health and illness (Carver & Morley 2017) ; Crichton et al. 2017; Scrutton 2017), clinical communication and language barriers (Naldemirci et al. 2020; Peled 2018), as well as medicalisation (Wardrope 2015). At the institutional level, the concept has been applied to issues such as psychiatric classification systems (Bueter 2019), treatment protocols for intersex people (Merrick 2019), and evidence-based healthcare policy (Michaels 2021; Moes et al. 2020). However, epistemic injustices in other domains such as scientific knowledge production (Grasswick 2017) or in connection with the use of big data technologies (Origgi and Ciranna 2017) can also have an impact on healthcare. Regarding big data, for example, the increasing digitalisation of medicine and healthcare can aggravate existing forms of pathocentric epistemic injustices if patients have to compete with technologies for credibility (Bennett and Os 2020).

### Considering solidarity

Many authors who have analysed epistemic injustices in healthcare have also made suggestions on how to overcome them and achieve epistemic justice. Some argue, for example, that healthcare professionals ought to become better aware of how their behaviour contributes to pathocentric epistemic injustices and correct these by cultivating virtues such as epistemic humility (e.g. Buchman et al. 2017; Peled 2018; Wardrope 2015) or that justice is fostered by healthcare professionals fulfilling their epistemic duties such as eliciting patients’ experiential knowledge (Drożdżowicz 2021). Others, in their proposals to counteract epistemic injustices, emphasise the collective dimension of generating knowledge in healthcare. Byrne (2020, p. 378), for example, contends that understanding illness is ‘deeply collaborative’, and Carel and Kidd 2014) , p. 537) emphasise that they understand ‘the quest for knowledge as a shared enterprise’. Although authors suggest that overcoming epistemic injustices is a collaborative endeavour, they do not further conceptualise the type of collective knowledge practices necessary to achieve epistemic justice.

In this article, I propose that the concept of solidarity—which, so far, has received no attention in connection with epistemic injustice and justice in healthcare—can fill this gap. Certain collective knowledge practices (specified in later discussions), I suggest, can be understood as epistemic solidarity, which can contribute to overcoming epistemic injustices in healthcare and—independently from contributing to epistemic justice—can also play a role in the generation of medical knowledge more generally. With the concept of solidarity we can capture those practices that challenge existing power relations in knowledge generation and resulting injustices as well as, more generally, grasp collective epistemic practices that aim to produce knowledge with a common benefit. Suggestions that others have made to overcome epistemic injustices can thus be important components of epistemic solidarity—in practice as well as conceptually. However, in contrast to calls for healthcare professionals to become more virtuous and dutiful, as well as in contrast to related concepts such as cooperation or altruism, I understand solidarity to be a political practice insofar as it challenges the distribution of epistemic power and is based on a certain ideal regarding the question of how we want to live together as knowers.

To discuss the role of epistemic solidarity in healthcare, I draw on the rich literature on ‘general’ solidarity in bioethics and beyond. Over the last decade, solidarity—and in particular Barbara Prainsack’s and Alena Buyx’s work on the concept (Prainsack and Buyx (Prainsack 2012) a); Prainsack and Buyx 2017) ; Prainsack and Buyx 2016; Prainsack 2018; Prainsack 2020)—has been widely discussed (Dawson and Verweij 2012) ; Kolers 2021; Prainsack and Buyx 2012b). Prainsack’s and Buyx’s notion of solidarity has been applied and critically assessed in connection with issues such as national healthcare systems (West-Oram 2018a), public health (Krishnamurthy 2013), unrestricted access to healthcare services (Gheaus 2016), care (Jennings 2018), responsibility (Davies and Savulescu 2019), genomics and precision medicine (Van Hoyweghen and Aarden 2021), data-driven medicine (Hummel and Braun 2020), refugee healthcare (West-Oram 2018b), and the COVID-19 pandemic (Johnson 2020; West-Oram 2021). Connected, but more specifically, others have discussed the relationship between justice and solidarity in healthcare and beyond. This line of literature argues that justice and solidarity are two equally important and complementary values (Ter Meulen 2015, 2016, 2017), but also that the experience of injustice can lead to practices of solidarity, which can contribute to the achievement of justice (Gould 2018; Tava 2021).

Not only conceptual considerations but also empirical examples highlight the relevance of solidarity in overcoming epistemic injustices. For example, that patients today experience epistemic injustice to a lesser extent than in the past is a situation (among others) resulting from patients organising in patient groups and collectives in which they fought to be seen, heard, and taken seriously (Britten and Maguire 2016) ; Brown et al. 2010). To an important extent, it was patients’ collective activism—and the solidarity among patients in which this activism was grounded and the result of—that instigated institutional change, and it also helped patients make sense of and express their illness experiences (i.e. to counteract hermeneutical injustices; Klawiter 2004) and to speak up in unjust interactions with physicians (i.e. to counteract testimonial injustices; Brashers et al. 2000). Although this is an example of how ‘general’ solidarity can contribute to epistemic justice in healthcare, my suggestion is to also account for epistemic solidarity proper and the role it can play in overcoming these injustices.

This article aims to draw attention to practices of epistemic solidarity, specifically in the context of healthcare; it aims to promote discussions on the concept, how solidarity can contribute to overcoming epistemic injustices, and the role it can play in knowledge generation in medicine more generally. In the remainder of this article, I first present and discuss Prainsack’s and Buyx’s concept of solidarity and address, by drawing on additional literature, solidarity’s relation to justice and injustice (Sect. 2). Subsequently, and based on this general discussion of solidarity, I propose a conceptualisation of epistemic solidarity and discuss it against the background of the existing literature on this concept (Sect. 3). Finally, I apply the concept of epistemic solidarity to two instances of collective knowledge production in healthcare, namely medical data sharing by patients and doctors’ engagement with patients to understand their afflictions (Sect. 4).

## What is solidarity?

### Defining the concept

Over the last decade, Barbara Prainsack’s and Alena Buyx’s work has invigorated the discussion on the role of solidarity in medicine and healthcare (Prainsack and Buyx 2012a; Prainsack and Buyx 2017) ; Prainsack and Buyx 2016; Prainsack 2018; Prainsack 2020). I draw on their conceptualisation as the basis for my discussion of epistemic solidarity because their definition of solidarity was developed in the context of medicine and has already been applied to various healthcare-related issues. Prainsack and Buyx 2017) , p. 52) define solidarity as ‘an enacted commitment to carry “costs” (financial, social, emotional or otherwise) to assist others with whom a person or persons recognise similarity in a relevant respect’. Their definition consists of three elements: The first is that solidarity is an enacted commitment, which means a concrete practice. Feeling sympathy for somebody in need of support is not solidarity; instead, the feeling must be expressed through action. However, with the defining element being its concrete realisation, solidarity can also be a feature of institutions, as Prainsack and Buyx point out. The second element of the definition is the type of actions that solidarity demands. Solidarity refers to assisting others and, in doing so, bearing costs. This means spending something (such as money or time) or accepting unpleasant emotions or a disadvantage (such as a risk to oneself) as part of helping others. If I act in solidarity with someone, I might benefit from doing so, immediately or in the long run. What sets solidarity apart from practices such as cooperation, however, is that accruing a personal benefit is not my primary motivation; accepting certain costs for myself is an expression thereof.

The third element of the definition concerns whom someone is willing to support in this manner. Prainsack and Buyx specify that solidarity practices are directed at others, with whom the ones who act in solidarity recognise a relevant similarity. They emphasise that solidarity is based on similarity as a means of distinguishing practices of solidarity from charity and altruism, which are based on differences between the ones who give and the ones who receive; the giver has resources and does not need help, whereas the recipient has insufficient resources and needs help. Solidarity, however, is based on the recognition that even if its practitioners might not need help now, they are in principle dependent on others in ways similar to those they are supporting. In contrast to related concepts, the specificity of solidarity is that it is not motivated by self-interest (unlike cooperation, for example), but it is also not only based on regard for others (unlike altruism, for example). Instead, solidarity rests on a relational understanding of personhood and recognises that self- and other-regarding motivations often cannot be neatly separated but instead are intertwined.

Two further aspects of solidarity should be briefly addressed. The first is how to deal with cases in which people, according to this definition, practice solidarity but the practice takes an unwanted form. For example, can we speak of solidarity if it occurs among a group of racist people whose mutual support is based on their prejudices and hatred and ultimately aims at excluding and harming others? Prainsack and Buyx address this question and contend that there exist both desirable and undesirable forms of solidarity. They argue, however, that desirable forms of solidarity can be distinguished from undesirable solidarity through the element of ‘sensible inclusivity’. This means that desirable forms of solidarity ‘avoid that people who are excluded [from these practices of solidarity] experience disadvantages, which substantially constrain their lives (respectively their *capabilities*)’ (Prainsack and Buyx 2016, p. 94, own translation). With their general definition, Prainsack and Buyx propose a descriptive understanding of solidarity, and through the argument of ‘sensible inclusivity’, they add a normative element to distinguish between desirable and undesirable solidarity. In my understanding, however, it is a crucial feature of solidarity that the practice thereof must not undermine other values such as inclusivity or justice.

Second, can all practices that exhibit the three elements and are normatively desirable be characterised as solidarity? Although Prainsack and Buyx do not address this question, I think that solidarity should not only be delineated from concepts such as cooperation and altruism and normatively undesirable forms of support but also from simply helping others. In my understanding, the term solidarity should be reserved for practices that are in some sense political; by this, I mean that they intervene in and aim to change a particular aspect of the social world (such as existing power relations) or have social implications beyond the particular situation in which they take place. Hence, in accord with Prainsack and Buyx (2017, p. 52), I understand solidarity as ‘an enacted commitment to carry “costs” (financial, social, emotional or otherwise) to assist others with whom a person or persons recognise similarity in a relevant respect’. However, and here I deviate from their definition, I only consider normatively desirable and transformative practices to qualify as solidarity. Such an understanding is also necessary for conceptualising solidarity’s relationship with justice and injustice, to which I turn in the following.

### Solidarity and its relation to (in-)justice

Justice and solidarity are widely understood to be complementary values and closely entwined; they are two sides of the same coin as Jürgen Habermas (1990) famously put it. Drawing on Habermas and others working in the tradition of Critical Theory, Ter Meulen (2015; 2016; 2017) discusses the relationship between solidarity and justice specifically in the context of healthcare. He asserts that (liberal) notions of justice tend to focus on people’s individual rights and duties and are based on an understanding of people as autonomous, self-interested individuals who negotiate their interests and balance them against each other, thereby abstracting from their interdependencies and relationships. By contrast, solidarity recognises that people are in many respects dependent on each other and acknowledges the reciprocal obligations and responsibilities they have towards others. Solidarity is based on an expression of the commitment to others’ well-being, which is considered to be intertwined with one’s own well-being. In this sense, solidarity is distinguished from liberal notions of justice by its focus on the relationality of human beings.

Ter Meulen argues that both values—justice and solidarity—should guide healthcare policy and practice; however, in his account, justice is the currently dominant value, which has potentially negative implications:

‘The increased emphasis on the concept of justice to analyse distributions of benefits and burdens in health and social care has the risk of a diminishing of attention for the personal bonds and commitments on the level of care practices. This may result in an impoverishment of the relations in health care which are fundamentally based on benevolence and commitment to the well-being of the other’ (2015, p. 18).

According to Ter Meulen (2016, p. 518), solidarity is necessary as a counterbalance to justice and serves ‘to promote the relational aspects of health care which are ignored and can even be undermined by policies based on justice only’. Although solidarity is complementary to justice and functions as a corrective, its promotion does not mean that solidarity should be considered an alternative to justice or that justice should be rejected.

Ter Meulen argues that solidarity, like justice, is an end in itself, but others have proposed to conceptualise solidarity through its connection to injustice and its relevance for achieving justice. Tava (2021) proposes such an alternative account of solidarity, drawing on (among others) Sally Scholz’s (2008) work, which understands solidarity as resulting from experiences of injustice and being directed towards social change. Based on such an understanding of solidarity (sometimes referred to as political solidarity), Tava questions the assumption that solidarity merely arises from reciprocal obligations and responsibilities people have towards each other. Instead, he argues that solidarity is often a reaction to injustice and that a ‘more substantive trail towards solidarity can be blazed if we look at its connection with injustice’ (Tava 2021, p. 1). In the context of healthcare such an understanding of solidarity has been more specifically promoted by Gould (2018). She contends that current discussions of solidarity in healthcare do not pay enough attention to structural injustices; yet, doing so would be crucial to understand the emergence of solidarity and the contexts and issues that engender solidaristic practices. Gould contends that analysing solidarity must involve an analysis of unjust healthcare institutions and practices and how they ‘generate, frame and motivate new solidarity movements to address these injustices’ (Gould 2018, p. 547). In this understanding, practices of solidarity result from and aim to counteract injustices; thus, solidarity is vital for achieving justice, in the sense that overcoming injustices often hinges on collective action and mutual support among those who suffer injustices and their allies.

Based on this brief discussion on the relationship between solidarity, injustice, and justice, we can summarise, first, that solidarity often arises in unjust situations and contexts and can be a critical component of achieving justice. This, however, is not to say that injustices necessarily lead to solidarity or that solidarity is the only means to achieve justice. Second, and returning to Ter Meulen’s argument, solidarity is not only instrumental for creating justice but is also—like justice—a moral value in itself. This is because solidarity acknowledges the reciprocal obligations and responsibilities that are part of people’s lifeworlds and thereby complements liberal notions of justice, which focus on people’s individual rights and duties. These two dimensions of solidarity, however, can also be related because practices to overcome injustices are often grounded in obligations and responsibilities people have towards each other. This is also why Ter Meulen argues that even though justice and solidarity ideally complement each other, solidarity is the more fundamental value because it constitutes the moral basis for justice.

## What is epistemic solidarity?

The main objective of this article is to transfer the concept of solidarity and considerations regarding solidarity’s relation with justice and injustice to knowledge practices. On the basis of Prainsack’s and Buyx’s definition of solidarity and the specifications outlined in the preceding discussion, I suggest defining epistemic solidarity as practices of supporting others (with whom one recognises similarity in a relevant aspect) as knowers. To qualify as solidarity, these practices must involve particular costs (such as spending time, giving up a privilege, or accepting risk for oneself). Furthermore, drawing on the literature that discusses solidarity and its relation with injustice and justice, epistemic solidarity, just like ‘general’ solidarity, can be of value in and of itself or a means to overcome epistemic injustices.

The concepts of epistemic injustice and justice are well established, but epistemic solidarity has received much less attention, even though social epistemologists have addressed the manifold ways in which we are dependent on others as knowers. Pointing to the collective dimension of knowledge generation, Sandford Goldberg, for example, states that ‘not only does the individual knowledge-seeker depend on the compliance of the world, what is more, this dependence includes an ineliminable dependence on other knowledge-seekers’ (Goldberg 2010, p. vii). He and others have asserted that because we need others in order to know, we engage in collaborative knowledge practices such as epistemic collaborations (Andersen and Wagenknecht 2013) or the division of epistemic labour (Goldberg 2011). Not all collective knowledge practices, however, take the form of ‘collaborations’ or ‘labour’. I suggest that those collective knowledge practices that consist of supporting others as knowers by accepting certain costs for oneself but that also aim at social transformation can be understood as epistemic solidarity. Consequently, the recognition that as knowers we are on many occasions dependent on each other can constitute the relevant similarity on which to base solidarity.

Despite several authors having already referred to epistemic solidarity, it is not an established concept. Before I apply the proposed definition of epistemic solidarity to some instances of collective knowledge practices in healthcare, I first discuss the differences and commonalities between my suggestion and existing accounts of epistemic solidarity. Most prominently, Medina (2013) refers to epistemic solidarity as a means ‘to think and believe together’. For Medina, epistemic solidarity constitutes the basis for developing capacities and contexts that enable and support a pluralism in perspectives, a diversity of voices, and a critical engagement with others’ experiences. Medina’s understanding of epistemic justice as represented in his vision of engaged diversity depends on solidaristic knowledge practice. Understanding epistemic solidarity as the precursor to epistemic justice, he stresses that practices of epistemic solidarity are the foundation of epistemic justice. In this sense, Medina’s argument overlaps with Ter Meulen’s (2016; 2017) claim that solidarity and justice are two complementary values but that solidarity provides the basis for justice. My understanding of epistemic solidarity corresponds with and builds on Medina’s notion but tries to further clarify it, as his account of the concept remains rather general.

Other authors who have provided more detailed accounts of epistemic solidarity have used the concept descriptively and without addressing solidarity’s relationship with injustice and justice. These authors address the collective dimension of knowledge production but do not—in my understanding—fully capture the particularities of solidarity. For Robert Goodin and Kai Spiekermann 2015), epistemic solidarity means organising to ‘produce correct beliefs’ (ibid., p. 439) and the ‘pooling [of] information with selected others’ (ibid., p. 440) to enable collective action. They apply epistemic solidarity to the context of voting behaviour and argue that—drawing on Marxist terminology—if the masses shared information about their true interests, they could overcome their false consciousness, which would enable them to act collectively and win elections against the elites. As I understand it, the example they give could be a case of epistemic solidarity, but the authors do not sufficiently distinguish those cases in which the pooling of information with selected others would classify as solidarity from those that would not. For example, the production of correct beliefs also occurs among intelligence agents who pool information to forestall collective action by the masses; such practice, I assume, Goodin and Spiekermann would not characterise as solidarity because their discussion of solidarity seems to link the concept to an emancipatory political project (although they do not specify this in their definition of epistemic solidarity).

Bird (2014) refers to epistemic solidarity to describe the cooperation among members of a research group and argues that collaboration among them is necessary to produce scientific knowledge. Such knowledge production indisputably hinges on epistemic collaboration, but generating knowledge with others is not automatically epistemic solidarity—whether people are being paid for generating knowledge and what type of knowledge they are generating are major considerations for the determination. As these accounts suggests, when employed as a descriptive concept, it is difficult to delineate epistemic solidarity from notions such as collaboration or cooperation. By contrast, Eric Wiland’s (2017) understanding of epistemic solidarity as the collective pursuit of knowledge that can yield common epistemic benefits is more convincing. For the case of scientific knowledge production, he argues that epistemic solidarity does not mean just generating knowledge together but—using voluntary participation in a citizen science project as an example—points to the relevance of *how* and *to what ends* people cooperate epistemically. The cooperation among researchers whose very job is to generate knowledge together is distinct from that of a group of people that voluntarily teams up for this end. Similarly, there is a difference in whether the knowledge produced mainly creates private gain (or even is used for oppressive ends) or whether it results in publicly valuable and accessible knowledge.

Wiland, however, is not only interested in epistemic solidarity in connection with the production of scientific knowledge but also in connection with moral testimony. When it comes to moral testimony, he explains, epistemic solidarity means deferring to those who are in a superior position to make a correct judgment. He gives the example of a woman telling her male companion that the behaviour of another man, which they had both just witnessed, was misogynistic. Wiland contends that the male companion who did not realise that the other man’s behaviour was wrong but defers to the woman’s judgement is demonstrating epistemic solidarity. He also asserts that deference on the part of the male companion can contribute to overcoming epistemic injustices that many women experience when addressing misogynistic behaviours. In Wiland’s understanding, exemplified in the two cases he discusses, epistemic solidarity contributes to the generation of a common epistemic good or epistemic justice. In this sense, his conception overlaps with my understanding of solidarity as political practice. In contrast to Wiland, I more strongly emphasize that solidarity comes with particular costs; however, both his examples contain this element of cost: Spending time (or other resources) to engage in a citizen science project as well as practising deference and giving up a hitherto held but (undue) privilege both constitute costs that the practitioners of solidarity in Wiland’s examples are willing to accept.

## Examples of epistemic solidarity in healthcare

I have proposed defining epistemic solidarity as practices of supporting others (with whom one recognises similarity in a relevant aspect) as knowers, which entail particular costs and have a political dimension. To return the discussion to the context of healthcare, I apply my proposed definition of epistemic solidarity to two examples, namely medical data sharing among patients and knowledge generation during doctor–patient encounters, and discuss what the concept can add to the analysis of these instances.

### Sharing medical data

Some authors have provided convincing analyses of how data sharing is a form of solidarity and how considering the principle of solidarity can contribute to better governance of medical databases (Hummel and Braun 2020) ; Machado and Silva 2015; Prainsack and Buyx (2017). I draw on these previous efforts, but I contend that data sharing (and other primarily knowledge-related activities) warrants a conceptualisation that more strongly emphasises the epistemic dimension of these practices.

How does epistemic solidarity feature in data sharing? At the individual level, for example, patients can practice epistemic solidarity by sharing their medical data, which they themselves or others have produced, and thereby contribute to the construction of medical databases. The analysis of such databases can yield new insights into health and disease, especially regarding diseases that remain poorly understood. This means that data sharing can yield benefits for the patients who share their data but also for all other people—at present or potentially in the future—suffering from the same disease. In this example, (potentially) suffering from the same disease and being dependent on others to understand it constitutes the relevant similarity between those who practice epistemic solidarity; the individual and common benefits of data sharing are thereby intrinsically entwined (Sharon 2017). Yet, the sharing of health data is also associated with certain risks such as data leaks, privacy violations, and potentially, even discrimination. Accepting these risks is the cost that people sharing their health data bear as part of their epistemic solidarity.

Risks and unintended damage are one category, but intended misuse is another. In this regard, Patrick Hummel and Matthias Braun (2020) contend that although health databases depend on practices of solidarity in the form of data sharing, large-scale collections of health data can also be used for unjust causes such as risk profiling and algorithmic discrimination. Therefore, they argue that solidarity and justice are in inherent tension, despite sometimes being complementary. In my understanding, however, if we take the complementary nature of justice and solidarity seriously (as discussed previously), data sharing in an unjust project or data sharing in contexts where patients’ rights are not respected does not qualify as epistemic solidarity, and post hoc misuse by a third party does not change the solidaristic nature of the practice of data sharing itself.

Prainsack and Buyx (2017) discuss how solidarity can both take place at the level of individual practice and also be a feature of institutions (the prototypical example being social health insurance); this also applies to the example of health databases. For example, the German Digital Healthcare Act (*Digitale-Versorgungs-Gesetz*), which was passed in 2019, regulates the collection of health data from electronic health records (EHR) as well as their use for research purposes. The act stipulates that EHR data are collected from everyone insured with a statutory health insurer but not from the approximately nine million people living in Germany who are privately insured. Due to the health database not containing data from millions of patients who are, on average, not only wealthier but also healthier than the patients with statutory health insurance (Hoebel et al. 2018; Hoffmann and Icks 2011), the results of research based on this database will inevitably be skewed because this segment of the population is not represented. Epistemic solidarity in its institutionalised form would mean changing the German Digital Healthcare Act to stipulate that all patients’ EHR data are included in the database. This means that privately insured patients would have to accept the risks that come with one’s health data being included in a digital database, but in doing so, they would be supporting the generation of more accurate knowledge regarding health and disease. This knowledge would benefit everybody (as both groups are similar in the sense that they can suffer from the same diseases) but particularly those people who currently experience worse health—typically, those with statutory health insurance.

Furthermore, it was shown that people with higher socioeconomic status enjoy more data-related rights and are more likely to be spared from data-based surveillance, which can be considered a form of injustice (Eubanks 2018). Hence, it would also be more just if patients in Germany—independent of their economic resources—were subject to identically strong regulations regarding the collection and use of medical data and attendant privacy protections. The current regulation concerning the use of data from EHR, in this sense, increases the injustice of a two-tier insurance system because people who can afford private insurance do not only enjoy better access to care (Klein and van dem Knesebeck 2016; Luque Ramos et al. 2018) and better health (Hoebel et al. 2018; Hoffmann and Icks 2011) but also better data protection than do those who cannot. Incorporating the principle of epistemic solidarity into laws regulating the use of EHR data, therefore, could 1) contribute to more accurate knowledge by circumventing the underrepresentation of certain population groups in these datasets and—in combination with adequate and equally good measures against data breaches for everyone—2) also be a means to overcome the unjust differences between how the health data of privately and publicly insured patients are handled.

### Doctor–patient encounters

As a second example—and linking epistemic solidarity with existing suggestions on how to overcome epistemic injustices in healthcare—I discuss knowledge generation in doctor–patient encounters According to Kidd and Carel (2014), epistemic injustices in healthcare settings occur when healthcare professionals exert unwarranted epistemic privilege. Insofar as healthcare professionals usually have more scientific knowledge about health and disease and particular knowledge resulting from the practice of medicine, in these domains, they are epistemically privileged. However, patients know their experience of illness, which is their epistemic privilege. Epistemic injustices thus occur when healthcare professionals disregard patients’ knowledge as relevant to understanding their afflictions and the care they receive and opt to instead act solely on the basis of their own knowledge and expertise. To overcome such epistemic injustices, some have called for doctors to exercise more epistemic humility and to acknowledge that patients’ epistemic contributions are essential to healthcare delivery (e.g. Buchman et al. 2017; Peled 2018; Wardrope 2015). For healthcare professionals to demonstrate epistemic humility means to acknowledge that the knowledge of both the doctor and the patient is relevant for a proper understanding of the patient’s condition.

Focusing on humility to overcome epistemic injustice is, however, limiting in two respects: First, humility is only an attitude, which still must be put into practice to take effect, and second, humility only contributes to testimonial justice. This is because humility implies that doctors efface themselves epistemically and thus provide patients sufficient time to speak or properly listen to them. Yet, often collective efforts are necessary to understand disease, but this view assumes that patients are already knowing and are only being deprived of the opportunity to share their knowledge or are just not being taken seriously. Being ill confers particular epistemic privileges on patients, such as knowing how their disease feels and what it means in the context of everyday life, but experiencing illness is also often unpleasant and can distort patients’ perceptions or ability to express themselves. Hence, they may require more from healthcare professionals than just being granted time to speak or being listened to. In their proposals for how to overcome epistemic injustices, other authors thus highlight the collaborative nature of generating knowledge in healthcare. They emphasise the importance of doctors’ inquiries (Byrne 2020) and how doctors should better fulfil their epistemic duty of eliciting patients’ experiential knowledge (Drożdżowicz 2021) through methods such as the use of a ‘phenomenological toolkit’, with which they could help patients to better make sense of and express experiences of illness (Carel and Kidd 2014). It is also argued that attempts to overcome epistemic injustices should acknowledge this collective dimension of knowledge generation because a narrow understanding of epistemic justice that fails to do so is ‘at the risk of obfuscating legitimate and potentially fruitful inquiry’ (Byrne 2020, p. 377). These suggestions acknowledge that collaboration, which is often necessary to understand patients’ afflictions, and doctors being more dutiful might contribute to epistemic justice; however, these suggestions are subject to certain limitations.

One limitation is that doctors ‘just’ fulfilling their duties might not be sufficient to overcome all healthcare-related epistemic injustices because such injustices do not only occur in individual doctor–patient encounters but are also ingrained in healthcare institutions and professional roles constructed around biomedicine and connected to healthcare institutions and professionals operating under often massive financial pressure and time constraints. If injustices are ingrained in institutions, overcoming them necessitates practices that go beyond what is envisaged and provided by these institutions; this means practices that go beyond or in some cases even conflict with doctors’ duties. In the context of doctor–patient encounters, epistemic solidarity, although consisting of the same practices, namely listening to and engaging with patients to generate knowledge about disease together, means doing these tasks in a manner exceeding the normal scope of doctors’ duties. This may mean that healthcare professionals take more time for patients than they normally would or more time than envisioned and suggested by their employer or by the routine procedures in their practice. This may imply seeing fewer patients (and accepting the associated costs, such as less revenue or conflicts with employers) or working longer hours. This is not to say that doctors *should*, for example, work longer hours out of solidarity with patients but rather that if they incur these costs because they engage in practices of supporting patients as knowers, it might be a case of epistemic solidarity. Epistemic humility, a sense of professional duty, and doctors recognizing the responsibility they have towards patients are prerequisites of epistemic solidarity. Yet, epistemic solidarity extends beyond them, and epistemic solidarity might be necessary to counteract the current distributions of epistemic power and resulting injustices, which are ingrained in and facilitated by current healthcare institutions and how they are organised.

Solidarity, as presented in a preceding discussion, also differs from other related practices, such as altruism or cooperation. Altruism, for example, is based on the differences between those who have and give something and those who receive it. Applied to knowledge practices in healthcare, altruism could mean that doctors share their knowledge with patients such as when explaining the somatic processes that led to the onset of their disease. Doctors know something which patients do not know, and doctors share this knowledge with patients. In so doing—and this might be how altruism extends beyond doctors’ professional duties—they might also take additional time in their patient interactions when, for example, they want to make sure that the patient has properly understood them. However, in contrast to solidarity, altruism, in my understanding, does not acknowledge that to understand disease both doctors’ and patients’ knowledge is relevant (i.e. the similarity that solidarity, but not altruism, is based on). Thus, if doctors acknowledge that both their own and patients’ epistemic involvement are necessary to fully understand patients’ afflictions and they engage in collective knowledge production during doctor–patient encounters, such a situation would not be a case of altruism.

Cooperation, in contrast to solidarity (and altruism), is not necessarily based on concern for others and can be purely instrumental for achieving one’s own goals. I would argue that in most doctor–patient encounters some form of epistemic cooperation takes place because doctors rely on information from patients to perform their job and provide every form of basic healthcare service. This cooperation, however, can take place to a minimal degree and without including patients’ knowledge to the full extent that would be necessary to provide truly adequate care. This also means that cooperation does not necessarily counteract epistemic injustices, although it can to a certain extent. Practices of epistemic solidarity in doctor–patient encounters, as discussed here, differ from cooperation because they are based on doctors accepting some form of cost to generate knowledge collaboratively with patients. Assuming such costs indicates doctors engaging with patients not primarily out of self-interest but out of care.

Epistemic solidarity in doctor–patient encounters can also be institutionalised—and ideally, it is. Institutionalised epistemic solidarity implies that the costs associated with listening to and engaging with patients are not solely borne by healthcare professionals but are distributed. For instance, health insurance could cover diagnostic and therapeutic conversations according to patients’ needs and reimburse doctors appropriately for conversations with patients more generally. In this case, costs are distributed among the collective pool of the insured. Although this could benefit all patients, it would be a particular service to people and groups who experience epistemic injustices in healthcare more frequently—for example, those who suffer from particularly complicated health issues or those who are less articulate or do not speak the dominant language well. Relatedly, but more generally, epistemic solidarity could be institutionalised if listening to and engaging with patients were counted as proper healthcare services and attributed a similar status—for example, in clinical guidelines—as biomedical interventions such as diagnostic tests, which would be particularly important in primary care settings. Yet another form of institutionalising epistemic solidarity would be to ensure a sufficient number of doctors can work free from time pressure and can, whenever necessary, take the time to properly engage with patients.

## Conclusion

In this article, based on Barbara Prainsack’s and Alena Buyx’s (2017) definition of solidarity and works addressing solidarity’s relationship with justice (Ter Meulen 2015, 2016, 2017) and injustice (Gould 2018; Tava 2021), I have proposed defining epistemic solidarity as practices of supporting others (who are similar in a relevant aspect) as knowers and thereby bearing certain costs. Additionally, to qualify as solidarity, these practices must be normatively desirable and in some manner productive or transformative, in the sense that they contribute to overcoming epistemic injustices or the creation of knowledge with a common benefit. In this sense, epistemic solidarity is a means of intervention and challenges current knowledge practices and what we know. To date, accounts of epistemic solidarity (except for Wiland 2017) have been rather general (Medina 2013) or have insufficiently demarcated solidarity from other concepts of collective knowledge production, particularly cooperation (Bird 2014; Goodin and Spiekermann 2015).

The last decade has seen a lively debate regarding ‘general’ solidarity’s role in bioethics and beyond, but solidarity has hitherto not been discussed in connection with epistemic injustice and justice in healthcare, and discussions concerning how to overcome epistemic injustices have so far neglected how solidarity, justice, and injustice are closely intertwined. As a corrective, the concept of epistemic solidarity can provide a complementary perspective on questions of knowledge-related injustice and how to overcome them. Consequently, epistemic solidarity goes beyond existing suggestions to counteract epistemic injustice, as it can address the epistemic injustice ingrained in medical and healthcare institutions. Furthermore, and beyond solidarity contributing to epistemic justice, solidarity is a valuable epistemic practice in and of itself. Epistemic solidarity recognises that as knowers, first, people are in many instances dependent on others and, second, that people do not only engage in collective knowledge practices on the basis of self-interest but also do so out of concern for others. In this sense, epistemic solidarity differs from concepts such as epistemic collaboration that account for epistemic dependences but fail to incorporate the knowledge practices that people engage in because they care about others.

The two examples of epistemic solidarity that I have discussed in this article are healthcare professionals’ efforts to engage with patients in the creation of knowledge concerning their conditions and patients sharing their medical data for research purposes. These examples demonstrate how epistemic solidarity can take place both at the individual level as well as on the level of institutions. Although I have focused here on the fields of medicine and healthcare, epistemic solidarity might be fruitfully applied in all areas in which epistemic injustices occur, where the goal is to achieve epistemic justice, and where people support others as knowers on the basis of a recognition of mutual dependence and because they care about them.

## Footnotes

1 The term ‘pathocentric epistemic injustices’ refers to epistemic injustices experienced by patients. Although pathocentric epistemic injustices are the focus of this paper, this does not mean that in the context of medicine and healthcare only patients can experience epistemic injustices.
